# The Abuse of Dental Charity

**Published:** 1887-10

**Authors:** 


ARTICLE IV.

### THE ABUSE OF DENTAL CHARITY.

Under the above caption the *Dental Review* publishes a very outspoken editorial, taking to task the dental colleges for abusing their opportunities by encouraging patronage from the wealthy or well-to-do classes. An outline of the writer's statements and criticisms includes the following ideas: The dental colleges advertise the rendering of services free, or at a merely nominal cost, placing no restrictions upon any who may choose to avail themselves of the privileges; the colleges in the large cities are, without exception, run as money-making institutions; their advertisements that fillings will be made at cost of material are false, their object being to entrap the unway. From 300 to 400 per cent. over and above the cost of material is ordinarily charged, and very often a handsome fee is exacted and paid. Handsomely-dressed ladies in sealskin sacques are among the frequenters of the operating rooms, etc., etc.

From these considerations (and other not quoted above), the writer concludes that the evil referred to urgently requires attention on the part of the various dental college faculties. He says: Private practice in Philadelphia has already been made to suffer seriously at the hands of the dental colleges, and the same evil threatents disaster to individual practitioners in other cities where colleges are located." The remedy suggested is to furnish "charity certificates" to public officials, physicians, dentists, clergymen, business men, and others who are willing and able to vouch for the patient's inability to pay a reasonable fee.

This is the first time we remember to have seen in print so unreserved an expression of opinion on this matter. The subject has been privately discussed among city dentists, to our knowledge. The statement that private practice has suffered seriously at the hands of any dental college would seem to require confirmation. Personally, we have never heard any particular dentist complain that his patrons have abandoned him in favor of the dental college. The fact that well-dressed persons have been seen among the patients at the dental college does not argue that some dentist has been robbed of a patron. By no means. As in many other matters, the question as regards dental services is, with certain penurious people, whether they shall get such services for a trifle or go without them. A good many dentists are so peculiarly constituted that they conceive a strong dislike for people who haggle about fees, so that their chief desire is to be well rid of them as quickly as possible. To cut the matter short, they perhaps recommend the dental college. No very extended inquiry would be needed to show that reputable practitioners occasionally send a poor but respectable-appearing applicant to the dental college, rather than leave him to drift into the hands of the cheap charlatan. The profession might perhaps do much to assist beginners in practice by sending poor but worthy applicants to them. But, as a matter of fact, most of the beginners want to be pretty well paid, too. Moreover, it should be remembered that the strictly pauper class can hardly be attracted in sufficient numbers to afford constant employment for a large class of dental students. They do not appreciate operative dentistry sufficiently to submit to the pain and tedium of operations, even when offered gratuitously. What is equally to the point, students are apt to be somewhat fastidious. A comely waiter-girl or a coy young chambermaid is regarded with more favor than a pock-marked pauper. The students select "matcrial" to suit their individual tastes from among acquaintances picked up during their winter's stay in boarding-houses. We used to do it ourselves.

If, then, we take the classes a grade or two above the paupers, we must incur the risk of respectable clothing, and perhaps submit to the occasional intrusion of a sealskin sacque. We have had occasion to notice that the sealskin sacques are sometimes cheap imitations worn by "ladies," as the appellation is used, but ladies whose charms of person and character are more gracefully exhibited in the kitchen than in the parlor.

Take it all in all, we doubt whether the dental colleges can be shown to be working detriment to the interests of any reputable practitioner. They may injure the quacks. The private practitioner is protected, in the nature of things, by several considerations. Pride deters many, even of the deserving poor, from recourse to the college infirmary. Even when the suggestion is made by the dentist, it is quite as often as otherwise regarded with disfavor. Many are unwilling to submit to the unavoidable publicity, others are well aware that the dental student is not the best conservator of time, and others again reflect that it is enough to endure their own sufferings at the hands of an operator, without being made liable to behold and participate in the sufferings of a score or two of fellow-unfortunates. These considerations are the profession's strong arm of defense against abuses in the dental colleges.

Again, in one view of the matter, the interests of the profession are constantly menaced by the work the dental colleges are doing in educating so many young men to become competitors with the present established dentists. A dozen or so new men swell the ranks of the city dentists with the close of every winter's term. It would be interesting to know what effect is wrought locally in such cities as Philadelphia, Chicago, St. Louis, and Cincinnati, by their entrance into practice. In the latter city we find by reference to the directory, about ninety dentists in active practice. Of this number, thirty-six are *recent* graduates of the Ohio Dental College. We include in this list only those who have offices of their own, not those who are attached to old establishments. Thirty-six new offices! Eleven years ago the then established dental offices in Cincinnati were only about forty in number. Possibly, something of this kind has been going on in Philadelphia.

Before concluding that the dental colleges of Philadelphia have done so much mischief to dental practice, it might be well to poison a score or two of these young, pestiferous interlopers, in order to determine exactly whence the evil proceeds. But if it can be shown that the dental colleges are exceeding their privileges, that they are aiming to secretly divert wealthy patronage to their doors, we stand ready to join in the hue and cry against the abuse. It appears, upon consideration, that no great amount of shrewdness would be required of unscrupulous managers to enable them to conduct the dental college as a vast monopoly. Let two or three shrewd, discreet men get control of the majority of stock shares and the rest is easily done. It would be no very difficult matter to convert the large, public operating room into a dozen or a score of private offices, thereby removing one of the great bugbears which frighten away timid, refined people. The students furnish all the labor, not only demanding no salaries, but really paying from $50 to $100 per term for the privilege of practicing and being instructed. Many of them are fine operators, and under the surveillance of the professors, the college would quickly gain a reputation for excellence and thoroughness of operations, such as might well alarm the private practitioner. Many of the colleges already enjoy such a reputation, well merited, too. It is a matter of no concern to the student that his labor brings him no pecuniary reward. Be that as it may, he cannot afford to quarrel with the authorities of the institution. Unquestionably, by a little tact and management, the dental colleges could thus be made to return large revenue to the managers. However, come to think of it, we are not aware that students are required by any present existing regulations to operate every day. We have it, however, on authority of two officers of a well-known dental college, that their operating rooms have been crowded during the entire collegiate year, keeplng a large class constantly busy. The uniform excellence of the work submitted for inspection gave evidence of plenty of practice on the part of the students. Still, some students are fine operators at the time of entering college.

Finally, we do not know that the colleges are sacredly pledged to the profession to confine their ministration to the very indigent or pauper classes. Inasmuch, however, as they look to the profession for countenance and support, it seems that deviation from what is presumed to be a proper cause, *i. e.*, avoiding whatever may injure private practice, may be regarded as justly censurable. Of course, no local practitioner could be expected to cherish and encourage an institution which, under guise of elevating the profession by properly qualifying entering members, stealthily invades the field which should be left to fair competition among outsiders.

---

